# Synergic effect of dielectric barrier discharge plasma and hydrodynamic cavitation on decolorization of methylene blue

**DOI:** 10.1371/journal.pone.0348118

**Published:** 2026-06-08

**Authors:** Ahmad Ahadi, Amirhossein Zali, Mohammad Ghorbanalilu, Hamid Ghomi

**Affiliations:** 1 Department of Physics, Shahid Beheshti University, Tehran, Iran; 2 Laser and Plasma Research Institute, Shahid Beheshti University, Tehran, Iran; Shahrekord University, IRAN, ISLAMIC REPUBLIC OF

## Abstract

The growing demand for efficient and sustainable wastewater treatment technologies has driven the development of advanced oxidation processes (AOPs) that combine multiple physical and chemical mechanisms. In this study, we introduce a novel hybrid system that integrates Dielectric Barrier Discharge (DBD) plasma with Hydrodynamic Cavitation (HDC) to achieve rapid decolorization of Methylene Blue (MB) in both distilled and tap water. Experimental conditions included applied AC voltages ranging from 7 to 12 kV and initial MB concentrations of 10–40 mg/L, with samples collected over treatment times up to 15 minutes. Complete decolorization was consistently achieved within 15 minutes, with ~80% removal typically occurring in less than 5 minutes. Notably, the energy utilization efficiency for the most concentrated solution (40 mg/L) at full decolorization reached 4.6 g/kWh, surpassing previously reported values (<4 g/kWh). Direct plasma–cavitation coupling (direct DBD-HDC mode) outperformed the indirect configuration, highlighting the synergistic effects of simultaneous microbubble cavitation and plasma-generated reactive oxygen species (ROS). Optical Emission Spectroscopy (OES) confirmed the presence of key oxidants such as hydroxyl radicals (•OH), which play a dominant role in dye degradation. These findings establish DBD-HDC hybridization as a powerful and energy-efficient AOP, with strong potential for scalable application in wastewater treatment and other environmental remediation processes.

## 1. Introduction

In July 2010, the United Nations officially recognized access to safe drinking water as a fundamental human right [1]. However, the rapid growth of global population, industrialization, and agricultural activities has intensified the demand for freshwater resources, resulting in severe stress on water availability and quality [2,3]. Consequently, water recycling and reuse have become critical components of sustainable water management strategies. Among various sources of aquatic pollution, the textile industry stands out due to its discharge of large volumes of dye-contaminated effluents. These dyes are chemically stable, non-biodegradable, and highly visible even at trace concentrations, posing serious ecological and health risks [4].

Conventional treatment technologies—including physical, chemical, and biological processes—are efficient in removing suspended solids and pathogens [5], but they are often ineffective in degrading persistent organic pollutants (POPs). These compounds, typically found in industrial wastewater, are chemically stable, highly mobile, and resistant to biodegradation, and are associated with neurological, gastrointestinal, and carcinogenic effects [6]. Therefore, the development of advanced treatment processes capable of mineralizing these recalcitrant pollutants is essential for ensuring both environmental and public health protection.

Advanced oxidation processes (AOPs) have emerged as promising techniques for the degradation of refractory organic contaminants through the generation of highly reactive species, particularly hydroxyl radicals (•OH) [7]. Among AOPs, plasma-based systems—especially dielectric barrier discharge (DBD) plasma at atmospheric pressure—offer unique advantages, including room-temperature operation, minimal chemical consumption, and in situ generation of highly reactive species such as •OH, O_3_, H_2_O_2_, and UV radiation [8–10]. In water treatment, these species are produced locally at the gas–liquid interface or within microbubbles formed in the liquid phase [9], allowing efficient decomposition of POPs into harmless products (CO_2_ and H_2_O) without secondary contamination [11]. Moreover, the use of ambient air as the feed gas enhances safety and reduces operational costs [12]. Despite these benefits, the short lifetimes and limited solubility of plasma-generated radicals restrict overall oxidation efficiency, emphasizing the need for enhanced gas–liquid mass transfer.

Integration of microbubbles into plasma reactors has emerged as a promising strategy to overcome this limitation by increasing the interfacial area and improving mass transfer [13]. Microbubbles facilitate the diffusion of short-lived species such as O, •OH, O_3_, and NO into the bulk liquid, enhancing the degradation of persistent organic contaminants [14,15]. Additionally, bubble collapse promotes turbulence and uniform dispersion of reactive intermediates throughout the solution, thereby accelerating reaction kinetics. Plasma reactors with bubble injection have demonstrated superior pollutant removal performance compared with conventional DBD configurations [16,17].

Hydrodynamic cavitation (HDC) is another advanced technique that exploits rapid formation and implosive collapse of vapor cavities under fluctuating pressure fields [18]. The collapse of cavitation bubbles generates localized ‘hot spots’ with extreme temperatures (1000–5000 K) and pressures (100–5000 Pa), inducing intense shear and shock effects that facilitate free radical formation and pollutant oxidation [19]. HDC has proven effective for degrading dyes, pesticides, and pharmaceuticals [20–22], as well as for disinfection of microorganisms including Microcystis aeruginosa, Legionella pneumophila, and Rotavirus [23–25]. Among different HDC configurations, Venturi-based systems outperform orifice designs due to more stable flow and higher cavitation intensity [26,27].

Despite the individual effectiveness of Non-Thermal Plasma (NTP) and HDC in degrading organic pollutants, each method faces specific limitations. Plasma systems often suffer from mass transfer limitations at the gas-liquid interface, while HDC alone may require long treatment times for complete mineralization. To overcome these challenges, the integration of plasma with cavitation has emerged as a promising Advanced Oxidation Process (AOP). This hybrid approach leverages the synergistic effect of both technologies: HDC enhances the contact surface and mass transfer through the generation and collapse of microbubbles, while plasma supplies high concentrations of reactive oxygen and nitrogen species (RONS).

Previous studies have demonstrated the potential of this integration; for instance, [28] showed that combining an orifice-based cavitation with a plasma discharge significantly improved the degradation rate of recalcitrant dyes. Similarly, research by [29] indicated that the shockwaves and micro-jets from cavitation can physically break down molecular structures, making them more susceptible to chemical attack by plasma-generated radicals like •OH and O_3_. Therefore, the synergy between these two physical and chemical phenomena can lead to higher energy efficiency and faster decolorization.

In recent years, hybrid advanced oxidation processes combining non-thermal plasma with cavitation have attracted increasing attention, as both techniques can generate highly reactive oxidizing species and can potentially reinforce each other. Cavitation (acoustic or hydrodynamic) can intensify mass transfer, promote mixing, and create localized hot spots and radical chemistry during bubble collapse, while plasma provides additional reactive oxygen and nitrogen species at the gas–liquid interface. Accordingly, several studies have explored plasma–cavitation integrations for pollutant degradation and disinfection, reporting faster kinetics and/or improved energy efficiency compared with standalone processes [28,29]. Nevertheless, the number of studies addressing integrated reactor designs and quantifying synergy under well-defined hydrodynamic conditions remains limited, particularly for compact coaxial configurations suitable for scale-up.

In this study, a hybrid DBD-HDC reactor was developed in which plasma discharge occurs on spray region, allowing continuous operation and enhanced radical production. Theoretical analysis based on bubble dynamics and Paschen’s law suggests that the transient low-pressure environment inside collapsing bubbles promotes intra- and inter-bubble electrical discharges [30], leading to intensified radical formation.

Hydroxyl radicals (•OH), generated through water dissociation during bubble collapse (Eq. 1), and additional •OH formed via ozone-mediated reactions at the gas–liquid interface (Eq. 2), act as the principal oxidants responsible for pollutant degradation, as below:


$$\begin{array}{c} H_{\text{2}}O\overset{HDC}{\to }\;\cdot H+\;\cdot OH \end{array}$$ (1)

$$\begin{array}{c} \text{3}O_{\text{3}}+\cdot OH+H^{+}\to \text{2}\cdot OH+\text{4}O_{\text{2}} \end{array}$$ (2)

Therefore, this work aims to (i) design and evaluate a hybrid DBD-HDC reactor for the degradation of organic pollutants and effective microbial inactivation, (ii) analyze the synergistic interactions between plasma and cavitation-induced radicals, and (iii) assess energy efficiency and process scalability. The proposed hybrid system offers a potential pathway toward high-performance, low-energy, and sustainable wastewater treatment technologies.

## 2. Materials and methods

### 2.1. Materials

Methylene Blue (MB), a heterocyclic thiazine-based cationic dye (C_16_H_18_N_3_ClS; 319.85 g/mol), was selected as the model organic pollutant. Analytical-grade MB (CI 52015) was used without further purification. Distilled water was prepared using a commercial reverse osmosis (RO) unit, while tap water was obtained from the local municipal water network. Both water types were stored under identical conditions prior to use to ensure consistency across experiments.

### 2.2. Preparation of MB solutions

A series of MB solutions with concentrations of 10, 20, 30, and 40 mg/L were prepared using both tap and distilled water. Two solution volumes—4 L and 8 L—were used for different test conditions. A precise amount of MB powder was weighed using an analytical balance and dissolved in 100 mL of the selected solvent in a volumetric flask. Initial dissolution was assisted by manual shaking and subsequently sonicated in an ultrasonic bath (Model: SONIC 4.5MX) to achieve complete dissolution. The stock solution was subsequently diluted with the same solvent to reach the desired concentration and volume. Each sample was subjected to plasma treatment for up to 15 minutes under various applied voltages. Aliquots were collected at 1, 2, 3, 5, 8, 11, and 15 minutes. All experiments were performed at an ambient temperature of 25 ± 1 °C and repeated in triplicate to ensure reproducibility and statistical reliability.

### 2.3. Chemical analysis

The pH of each sample was measured using a calibrated Sartorius PB-11 pH meter equipped with a KCl-filled combination electrode and automatic temperature compensation. Electrical conductivity (EC) was determined using a NeoMet CP-500L conductivity meter calibrated with a standard K = 1 solution. UV–Visible spectra were recorded on a Biochrom Ultrospec 2100 Pro spectrophotometer over the wavelength range of 190–900 nm, with a photometric accuracy of ±0.003 A. Prior to analysis, all samples were sonicated to ensure homogeneity. The MB mass was determined with a Sartorius TE214S analytical balance (Germany). All instruments were recalibrated before each measurement cycle to minimize systematic error.

Chemical oxygen demand (COD) was determined using the standard closed reflux dichromate titration method according to Standard Methods (5220C). Briefly, liquid samples were digested in a strongly acidic medium containing potassium dichromate (K_2_Cr_2_O_2_) as the oxidizing agent and silver sulfate as catalyst. The sealed digestion mixture was heated at 150 °C for 2 h. After cooling to room temperature, the excess dichromate was titrated manually with standardized ferrous ammonium sulfate (FAS) solution using ferroin indicator as the endpoint detector. COD values were calculated based on the volume difference between blank and sample titrations and expressed as mg O_2_/L. All measurements were conducted in triplicate (n = 3) and the mean values were reported.

Total organic carbon (TOC) was measured using a LECO TOC-430 analyzer based on high-temperature catalytic combustion. Liquid samples were directly introduced into the combustion chamber, where organic carbon was oxidized to CO_2_ at elevated temperature in an oxygen-rich environment. The produced CO_2_ was quantified using an infrared detection system integrated into the instrument. Prior to analysis, inorganic carbon was removed by acidification and purging to ensure selective determination of organic carbon. Each sample was analyzed in triplicate and average values were reported.

Hydrogen peroxide was not detected in the treated samples at the time of COD analysis; therefore, no additional quenching step was required prior to titration.

### 2.4. Methylene blue (MB) degradation

The concentration of MB in the treated samples was determined via UV-Visible absorption spectroscopy. The characteristic absorbance peak of MB at λ = 664 nm was used for quantification. The dye concentration at each time point was calculated based on a previously established calibration curve (R^2^ > 0.999). The degradation efficiency, η (%), was evaluated according to Eq. (3):


$$\begin{array}{c} degradation\;efficiency\;(\eta )\;[\%]=\text{1}\text{0}\text{0}\times \frac{C_{\text{0}}-C_{t}}{C_{\text{0}}} \end{array}$$ (3)

where C_0_ (mg/L) and C_t_ (mg/L) denote the initial and instantaneous dye concentrations at time t, respectively.

To assess the energy performance of the DBD-HDC process, the energy utilization efficiency was calculated as follows:


$$\begin{array}{c} \gamma \left[\frac{g}{kWh}\right]=\frac{V\;[L]\times C_{\text{0}}\;[g/L]\times (\eta \;[\%]\times \frac{\text{1}}{\text{1}\text{0}\text{0}})]}{P\;[kW]\times t\;[h]} \end{array}$$ (4)

where V is the total treated solution volume, P is the electrical discharge power, and t is the plasma treatment duration. This parameter reflects the mass of dye degraded per unit of electrical energy consumed, thereby serving as a measure of system energy efficiency.

The reaction kinetics were analyzed assuming a pseudo-first-order model:


$$\begin{array}{c} k_{app}t=ln\left(\frac{C_{\text{0}}}{C_{t}}\right) \end{array}$$ (5)

The apparent rate constant (k_app_) was determined from the slope of the linear regression of $\text{ln}\left(\frac{{\text{C}}_{\text{0}}}{{\text{C}}_{\text{t}}}\right)$ versus time.

All regressions yielded correlation coefficients (R^2^) greater than 0.95, confirming the validity of the pseudo-first order assumption [31].

### 2.5. Plasma setups

#### 2.5.1. Reactor design and configuration.

To ensure system optimal performance, a precise and comprehensive system including all critical parameters, was designed and developed. A principal design challenge was the optimization of fluid injection from the HDC unit into the plasma region, while maintaining stable plasma generation and maximizing air suction into the reactor. These factors substantially influence system efficiency and necessitate extensive experimental validation. Subsequent efforts concentrated on sustaining effective plasma generation within the active zone to promote the formation of reactive species and their interaction with cavitation bubbles. Attaining this objective required careful optimization of key factors, including HDC unit and electrode geometry, material selection, and power-supply configuration.

The hybrid treatment system consists of a coaxial DBD reactor integrated with a HDC nozzle. The assembly is arranged in a concentric cylindrical configuration. The outermost layer comprises a stainless-steel cylindrical electrode (high voltage electrode) with an inner diameter (ID) of 30 mm and an outer diameter (OD) of 38 mm. Two different electrode lengths, 20 mm and 50 mm, were employed to investigate the effect of discharge volume. A quartz tube (OD 30 mm, ID 27 mm) serves as the dielectric barrier, positioned tightly within the outer electrode.

#### 2.5.2. Hydrodynamic cavitation nozzle and operation.

The HDC unit is an orifice-type nozzle precision-machined from stainless steel. The internal geometry is characterized by:

- Inlet section: Diameter (D_i_) of 6 mm and length of 20 mm.
- Throat (constriction): Diameter (d_th_) of 2.5 mm and length of 5 mm.
- Divergent section: A 7° half-angle expansion leading to an exit diameter of 3 mm.
- HDC nozzle OD: 23 mm, is placed coaxially inside the quartz tube.

**Fig 1:**
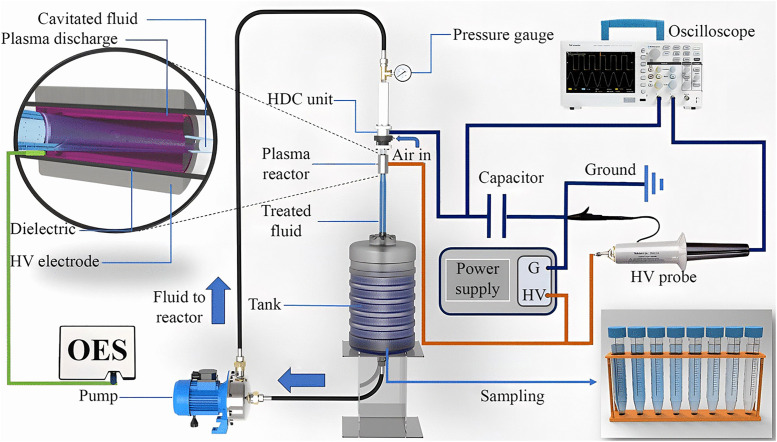
Direct HDC-DBD plasma setup schematic.

Fig 1 illustrates a cylindrical DBD plasma reactor integrated with a HDC generation system. The grounded electrode is realized by the cavitated fluid column flowing through the HDC unit. During operation, the fluid is pumped into the nozzle at an inlet pressure of approximately 780 kPa (measured via an analog pressure gauge). The passage of cavitating fluid into the tube induces a directed airflow toward the plasma zone, thereby supplying a continuous influx of oxidizing species and stabilizing the plasma temperature under steady-state conditions. As the fluid traverses the HDC unit, a progressive pressure drop occurs, driving entrainment of ambient air in the form of microbubbles until pressure equilibrium is established. This cavitation-assisted mechanism enhances interfacial mass transfer, intensifies turbulent mixing, and promotes effective coupling between plasma-generated species and the liquid phase. The HDC unit, coaxially integrated via a dedicated holder, comprises a metallic tube that facilitates bubble nucleation, growth, and detachment. Consequently, reactive species (RONS) generated within the plasma are efficiently transferred across the plasma–liquid interface through the microbubble environment. This configuration is hereinafter referred to as the direct reactor, as the plasma interacts directly with the flowing fluid, thereby maximizing the efficiency of plasma-induced chemical processes.

**Fig 2:**
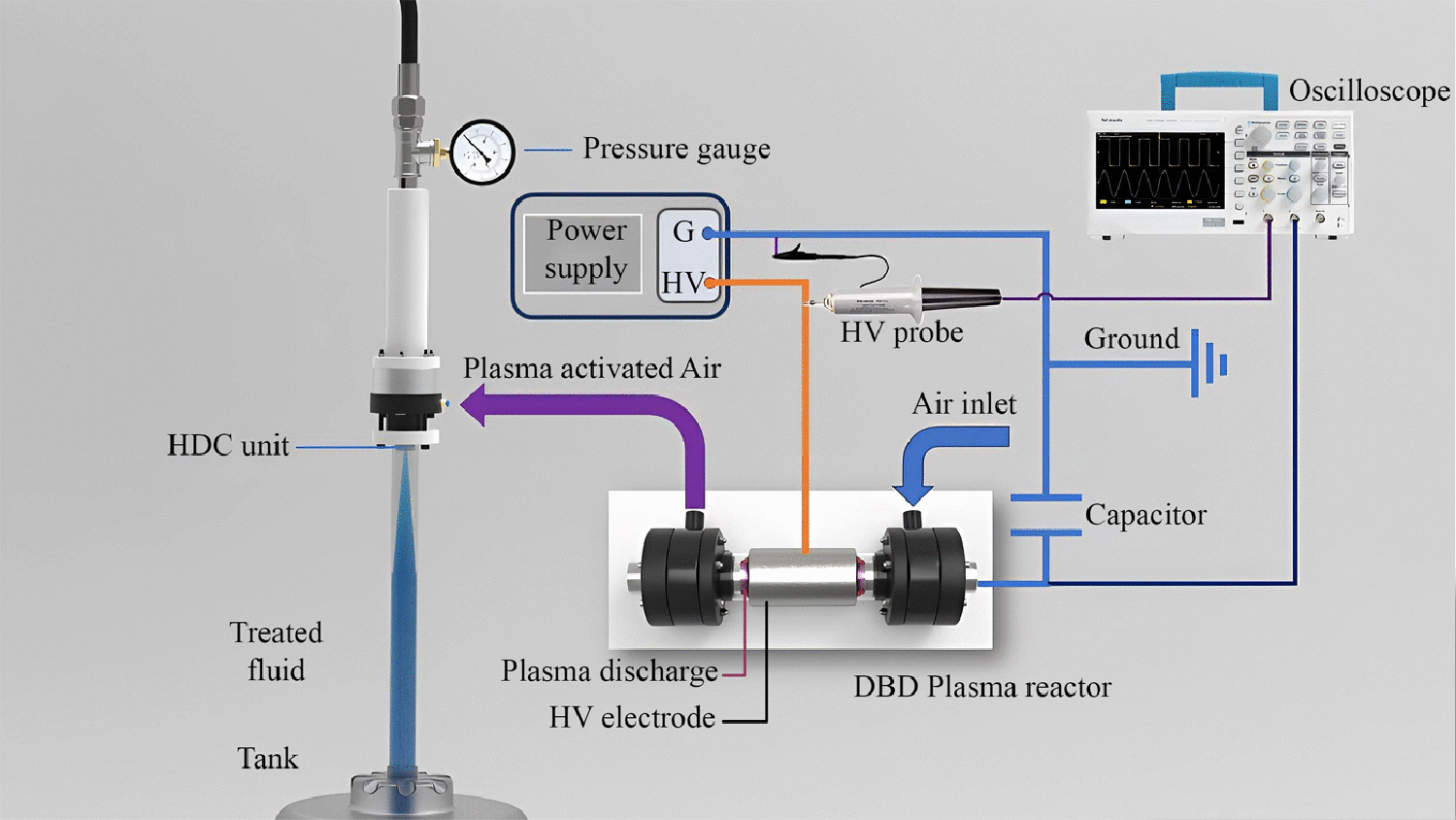
Indirect HDC-DBD plasma setup schematic.

To compare the performance of the direct effects of plasma on water, a second reactor was designed and constructed with identical specifications, dimensions, and dielectric–electrode gap as the first reactor. The reactive gas generated by this reactor was introduced into the flowing fluid through the HDC unit in the form of microbubbles. This setup is referred to as the indirect reactor (Fig 2).

To provide the necessary pressure and flow rate, an 800 kPa peripheral Pump was utilized. Its technical specifications include a rated voltage of 220–240 V, frequency of 50–60 Hz, current consumption of 14.5 A, maximum power of 0.75 kW, and a maximum flow rate range of 35 L/min. In the system setup, the solution was circulated from the reservoir into the device and returned after passing through the treatment section. Measured upstream water pressure of the HDC unit was 780 kPa, as indicated by an analogue pressure gauge. The flow of the HDC unit was 2.5–3 L/min, as indicated by an analogue flowmeter.

### 2.6. Physical discharge characterization

The plasma reactor was powered by a single-frequency switching AC power supply operating at 19.5 kHz. The output voltage was nominally sinusoidal; however, detailed observation revealed a distorted waveform with a sawtooth-like profile and superimposed high-frequency ripples.

The applied high voltage was measured using a TEKTRONIX P6015A high-voltage probe (1000:1 attenuation) connected to a TEKTRONIX TDS 2024B oscilloscope (200 MHz bandwidth). The maximum measured peak voltage (V_p_) was 15 kV, corresponding to a peak-to-peak voltage (V_pp_) of 30 kV, with the waveform oscillating between −15 kV and +15 kV. The probe tip was directly connected to the high-voltage electrode of the DBD reactor.

The discharge current was measured on the ground return path using a CC-65 AC/DC current clamp. The current probe output was simultaneously recorded by the oscilloscope along with the applied voltage signal to ensure proper phase alignment. Due to uncertainties associated with probe calibration and the complex nature of DBD current (including displacement current and filamentary micro-discharge components), the recorded current waveform is presented in arbitrary units and is used for qualitative analysis of discharge behavior only.

**Fig 3:**
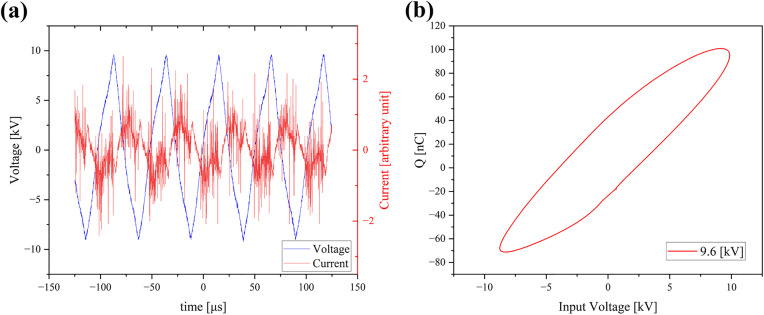
(a) Voltage-Current waveform in the direct HDC-DBD Plasma reactor. (b) Lissajous figure of direct HDC-DBD plasma reactor for 9.6 kV applied voltage.

As illustrated in Fig 3(a), the applied voltage to the system exhibits a well-defined sinusoidal waveform with an amplitude of approximately ±9.6 kV and a frequency of around 19.5 kHz, indicating the use of a high-voltage AC power supply, which is a common excitation method in DBD configurations. In contrast, the corresponding current waveform displays a highly nonlinear, pulsed behavior, characteristic of DBD plasmas. The current consists of a series of sharp, transient pulses occurring within each half-cycle of the applied voltage. These pulses result from dielectric breakdown across the inter-electrode gap and the formation of numerous transient micro-discharges in the plasma region. Each micro-discharge produces a short-duration (ns–μs) high-intensity current pulse, and the macroscopic current waveform represents the collective contribution of these micro-discharge events. As the applied voltage exceeds the critical breakdown threshold during each half-cycle, micro-discharges are randomly initiated at various locations across the dielectric gap, leading to the observed pulsed current behavior. The distribution of current pulses is relatively uniform and symmetrical, with a higher density of pulses occurring near the voltage peaks, indicating stable and consistent plasma generation under the given operating conditions.

### 2.7. Calculation of the power consumption

The electrical power consumption (P), as a function of the applied high voltage (V), reflects fundamental electrical properties of plasma reactors. Two prevalent methodologies exist for estimating the power consumed by a reactor, both involving the determination of the operating voltage and either the current or the charge (Q). These measurements are typically performed using a probe resistor or capacitor, respectively. We utilized the capacitor-based approach due to its capability to integrate the current passing through the reactor over time, thereby capturing all micro-discharge pulses with a suitably chosen probe capacitor. This method offers a superior signal-to-noise ratio compared to resistor-based measurements, as it effectively consolidates the discharge events. The operational voltage V and the charge Q are often plotted against each other in Q-V figures, also known as Lissajous figures. Power calculations were performed in accordance with the methodology described in reference [32]. This approach was systematically applied to evaluate the power characteristics under varying input voltage conditions. Power consumption was measured across a voltage range of 6.8 to 12 kV, utilizing a 22 nF polypropylene film capacitor (±5% tolerance, 4 kV rated voltage) connected in series with the reactor (see Figs 1 and 2). The voltage across the reference capacitor was measured using a 10 × passive probe connected to the oscilloscope. The measured peak voltage across the capacitor was 34.4 V. The results indicated that the power consumption of this reactor was in the range of 66–217 W. As an example, Fig 3(b) depicts the Lissajous figure of the direct HDC-DBD plasma reactor for an applied voltage of 9.6 kV.

### 2.8. Plasma reactor and OES

Optical Emission Spectroscopy (OES) of plasma flame is collected by TIDA spectrometer (Model UCS-G400). OES measurements were performed in the 200–1100 nm range at applied voltages between 6.8 and 12.0 kV. The spectral resolution of the spectrometer was 1 nm, and the integration time was fixed at 15,000 ms for all measurements to ensure comparability. Wavelength calibration was conducted using a mercury lamp prior to experiments. For conducting OES measurements, a dedicated structure was made that kept the plasma 5 cm distance to the spectrometer lens.

The emission intensities were measured as a function of wavelength, and the integrated intensity of the OH(A–X) band (304–308 nm) was calculated using numerical trapezoidal integration. Similarly, the N_2_ emission band (334–340 nm) was integrated as a reference species. The ratio ($I_{OH}/I_{N_{\text{2}}}$) was used as an indicator of reactive oxygen species production relative to nitrogen excitation.

### 2.9. CFD simulation

To ensure the onset of cavitation within the HDC unit, Computational Fluid Dynamics (CFD) simulations were conducted. Effective generation of hydroxyl radicals through hydrodynamic cavitation requires the formation of a sufficiently large vapor bubble region. Therefore, CFD analysis was employed to predict and visualize the development and spatial extent of the cavitation zone. Prior to physical fabrication, such predictive modeling is essential for evaluating cavitation intensity and optimizing geometric design parameters.

**Fig 4:**
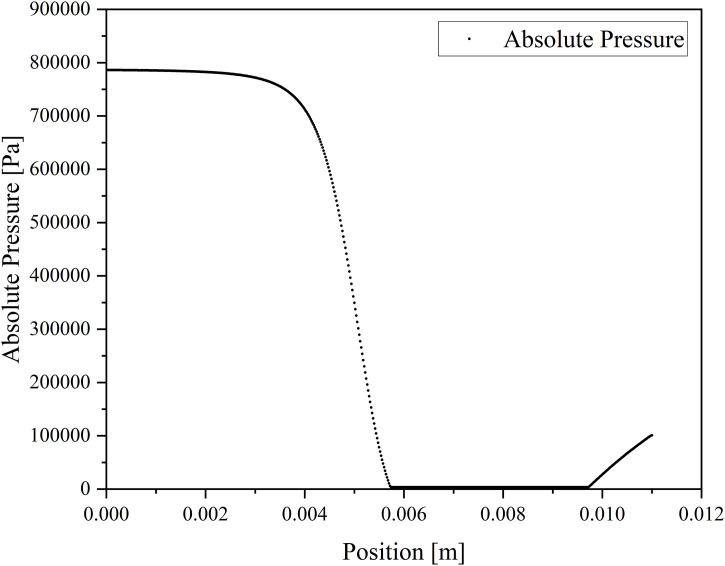
Absolute pressure per position profile of HDC unit for CFD simulation.

As illustrated in Fig 4, regions where the absolute pressure drops to or below the saturation vapor pressure of the liquid correspond to cavitation zones, with larger low-pressure regions indicating more intense vapor formation. All geometries were analyzed under identical boundary conditions: an inlet pressure of 780 kPa (as measured in the experimental setup) and a saturation vapor pressure of 3540 Pa for water. The nozzle geometry featured a divergence angle of 7°, an inlet diameter of 6 mm, and a throat diameter of 2.5 mm.

#### 2.9.1. Numerical method.

The numerical analysis of axisymmetric, incompressible, viscous, and homogeneous fluid flows is based on the Navier–Stokes equations. The incompressibility assumption is justified by the relatively low reciprocal of the squared speed of sound, indicating negligible density variations. The primary governing equations include the continuity and momentum equations. In the present study, a two-phase flow through an orifice-like nozzle is investigated, consisting of a bulk liquid phase and vapor-filled cavities. To accurately capture the interactions between the phases, a multiphase mixture model is adopted, with distinct sets of governing equations solved for each phase. The governing equations for both the mixture and the individual phases are implemented following the methodology outlined in reference [33], ensuring consistent treatment of interphase momentum and mass transfer. This approach allows for a detailed prediction of phase distribution, cavity dynamics, and the resulting hydrodynamic behavior within the nozzle.

#### 2.9.2. CFD model.

To investigate the influence of key parameters on the extent of cavitation, a CFD-based approach was adopted. The governing equations, including the continuity and momentum equations, were discretized using the finite volume method. Steady-state cavitation conditions were assumed as an approximation to reduce computational cost, with a no-slip velocity boundary condition imposed at the walls. The Schnerr–Sauer cavitation model was applied within a mixture framework to simulate multiphase flow, treating the liquid and vapor phases as interpenetrating continua. Turbulence effects were accounted for using the standard turbulence model.

Spatial discretization was performed using a first-order upwind scheme for density, momentum, vapor fraction, turbulent kinetic energy, and turbulent dissipation rate. Pressure–velocity coupling was handled with the SIMPLEC algorithm, and convergence was achieved when the residual for mass conservation reached. The simulation was carried out at a constant outlet pressure of 95 kPa. The computational mesh was constructed with an element size of 0.02 mm, a first-layer height of 0.005 mm, and 20 prism layers near the wall to adequately resolve boundary-layer effects.

#### 2.9.3. Results of CFD simulation.

Operational parameters such as inlet pressure and flow rate strongly influence the cavitation process. In this study, the effect of inlet pressure was investigated in the range of 400–1000 kPa using CFD simulations, with the outlet pressure fixed at 95 kPa. The velocities at the throat and the corresponding cavitation numbers for each inlet pressure are presented in Fig 4(b) and 4(c), facilitating the analysis of the hydrodynamic characteristics of the HDC unit.

The cavitation number ${\text{C}}_{\text{V}}$, is defined as:


$$\begin{array}{c} C_{V}=\frac{P_{\text{2}}-P_{V}}{\frac{\text{1}}{\text{2}}\rho \overset{\text{2}}{\underset{\text{0}}{v}}} \end{array}$$ (6)

Where P_2_ is the downstream pressure, P_V_ is the vapor pressure of the liquid, ρ is the fluid density, and $v_{\text{0}}$ is the velocity at the throat. Under sufficiently low downstream pressure, a smaller C_V_ (particularly below 1) enhances cavity formation. However, cavitation may also occur at C_V_ > 1 due to dissolved gases and heterogeneous nucleation. Previous studies have reported that the most effective range for wastewater treatment typically lies between C_V_ = 0.1 and 0.3.

Simulation results demonstrate that increasing the inlet pressure substantially extends the cavitation zone. For instance, an inlet pressure of 1 MPa yields the largest cavitation region, primarily due to the significant increase in throat velocity. Higher inlet pressures reduce C_V_, leading to a greater number of cavitation bubbles. Nevertheless, excessive reduction in C_V_ can trigger supercavitation, where a cavity cloud extends downstream and potentially obstructs flow. To avoid this phenomenon, the Venturi should be operated above the supercavitation threshold, thereby preventing choked cavitation conditions [34].

**Fig 5:**
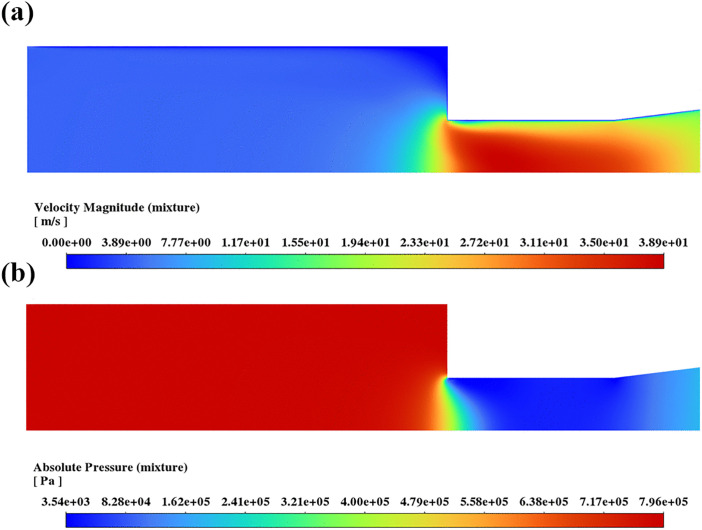
(a) velocity magnitude, (b) absolute pressure distribution of HDC unit for CFD simulation.

The simulations further indicate that the minimum pressure in the throat region reaches approximately 3530 Pa, which is close to the saturated vapor pressure of water at ambient temperature. This confirms the occurrence of continuous cavitation within the throat. As illustrated in Fig 5, the maximum velocity at the throat reaches 38.9 m/s, corresponding to a cavitation number of approximately 0.2 in the present system.

## 3. Result and discussion

### 3.1. OES analysis

#### 3.1.1. Identification of excited species.

To identify and characterize the reactive species generated in the plasma environment, OES was performed under various applied voltages (6.8, 8.4, 9.6, 10.6 and 12.0 kV). The dominant emission bands in the 330–400 nm region correspond to the second positive system of nitrogen (N_2_ (C^3^Πu → B^3^Πg)), with characteristic peaks at 337, 357, and 380 nm. These emissions are typical of atmospheric-pressure air plasmas and indicate efficient electron-impact excitation of nitrogen molecules [31,35].

In the near-UV region (290–310 nm), three distinct emission bands were observed at approximately 295.3, 302.4, and 306.3 nm. These are attributed to the vibrational transitions of the OH (A^2^Σ^+^ → X^2^Π) system:

- 295.3 nm → (1–0)
- 302.4 nm → (0–1)
- 306.3 nm → (0–0)

Due to the 1 nm spectral resolution, rotational fine structure could not be resolved; therefore, the observed features represent vibrational band envelopes [31].

**Fig 6:**
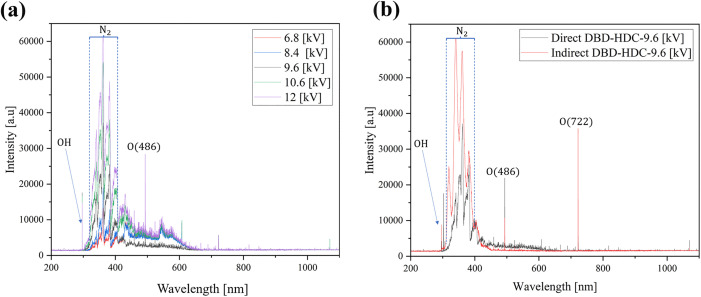
(a) Optical emission spectra (OES) of direct DBD-HDC plasma under various applied voltages (6.8, 8.4, 9.6, 10.6 and 12.0 kV). (b) OES of direct DBD-HDC and indirect DBD-HDC reactors at 9.6 kV.

Fig 6(b) presents the emission spectrum obtained at 9.6 kV under direct DBD-HDC and indirect DBD-HDC conditions. Similar spectral features were observed at other applied voltages; however, their relative intensities varied systematically with voltage (see Fig 5(a)). Weak atomic oxygen lines were detected at 722 nm, 749.8 nm, and 777.4 nm. The relatively low intensity of O emissions compared to OH and N_2_ is consistent with strong collisional quenching of excited O states under atmospheric-pressure conditions, where frequent collisions with N_2_ and O_2_ reduce radiative lifetimes [31,36].

#### 3.1.2. Quantitative comparison of reactive species.

To avoid purely qualitative interpretation, the integrated emission intensity of the OH (306 nm) band (304–308 nm) was calculated for each applied voltage:


$$\begin{array}{c} I_{OH}=\int_{\text{3}\text{0}\text{4}}^{\text{3}\text{0}\text{8}}I(\lambda )d\lambda \end{array}$$ (7)

Similarly, the N_2_ (337 nm) band was integrated over 334–340 nm and used as a reference to account for overall plasma excitation level:


$$\begin{array}{c} R_{OH}=\frac{I_{OH}}{I_{N_{\text{2}}(\text{3}\text{3}\text{7})}} \end{array}$$ (8)

Fig 1(a) presents the variation of R_OH_ as a function of applied voltage for both Direct and Indirect DBD-HDC configurations. In the Direct DBD–HDC reactor, as Fig 5 represents, I_OH_ increased from 6.8 to 9.6 kV, followed by a slight saturation or decrease till 12 kV. In contrast, the Indirect configuration exhibited lower absolute OH intensity at all voltages, and the growth rate with voltage was less pronounced.

This behavior suggests that in the Direct configuration, the spatial coincidence of plasma generation and cavitation bubble collapse enhances water vapor dissociation and local radical production. At higher voltages (12 kV), the increase in continuum emission indicates enhanced filamentary discharge and increased electron density, which may reduce the selectivity toward OH production.

#### 3.1.3. Correlation with methylene blue degradation.

The degradation efficiency of MB followed a similar voltage-dependent trend, with maximum removal observed near the voltage at which OH emission intensity peaked (9.6–10.6 kV).

**Fig 7:**
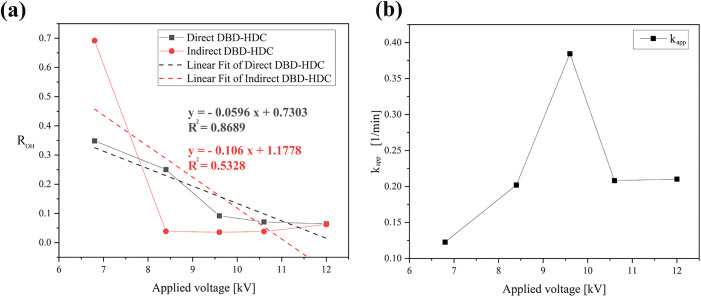
(a) variation of ROH as a function of applied voltage for both Direct and Indirect DBD-HDC configurations. (b) variation of kapp as a function of applied voltage for Direct DBD-HDC configuration.

To evaluate the correlation between plasma-phase OH production and liquid-phase degradation, the apparent degradation rate constant (k_app_) was plotted against I_OH_ (Fig 7(b)). A nonlinear correlation was observed, which was not supporting the role of plasma produced hydroxyl radicals as primary oxidative agents in the combined DBD–HDC process.

#### 3.1.4. Effect of liquid presence on plasma emission.

**Fig 8:**
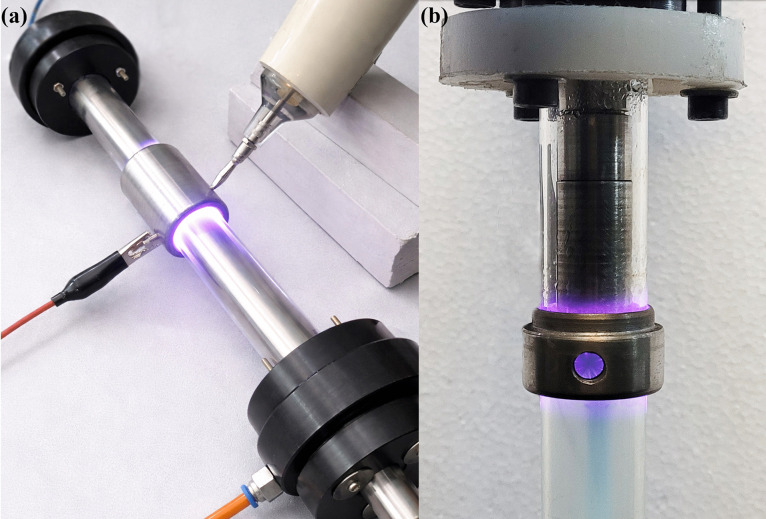
(a) Indirect DBD-HDC plasma reactor at 10.6 [kV] of applied voltage. (b) Direct DBD-HDC plasma reactor (2 cm electrode) at 9.6 [kV] of applied voltage.

Liquid occupied part of the reactor volume, may influence optical path length and emission intensity. Hence, we made a 5 mm diameter hole on each outer electrode (see Fig 8) to capture OES spectrum through it. However, all spectra were recorded under identical geometric and optical conditions. Therefore, the relative comparison between operating modes remains valid. The reduced gas volume likely enhances plasma–liquid interfacial interactions, contributing to increased radical transfer rather than merely altering optical detection.

Differences in emission intensity between direct and indirect treatments suggest that the diffusion of reactive species into the liquid phase modifies the optical signal. This behavior correlates well with the MB degradation trends discussed previously, supporting the role of OH and O radicals in the observed decomposition mechanism.

#### 3.1.5. Electrodes with different dimensions.

Two high-voltage electrodes of distinct geometries were tested. As shown in Fig 8(a), both configurations produced comparable degradation results, although the larger electrode (5 cm) demonstrated slightly superior efficiency and was therefore selected for subsequent experiments.

The maximum difference in MB degradation between the two electrode setups occurred during the initial 5 minutes of treatment, with a deviation of approximately 3%. This discrepancy diminished with longer exposure times, likely due to the approach toward steady-state plasma conditions, where the generation of reactive species saturates and both configurations converge in performance.

#### 3.1.6. Various applied voltages.

**Fig 9:**
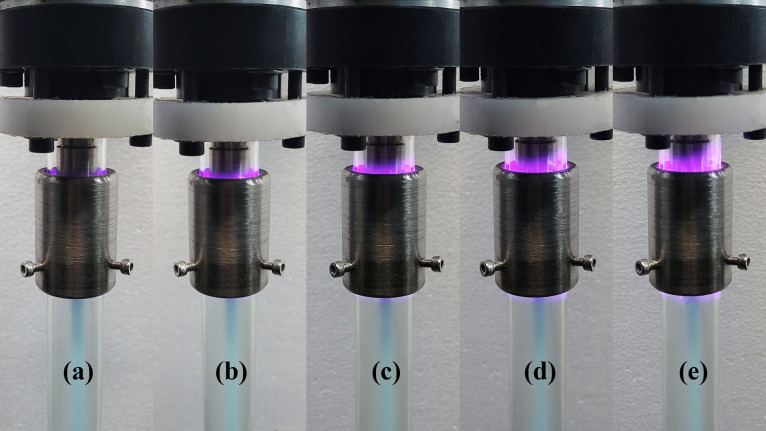
Direct HDC-DBD (5 cm electrode) system for (a) to (e) respectively 6.8, 8.4, 9.6, 10.6 and 12.0 [kV] of applied voltages.

**Fig 10:**
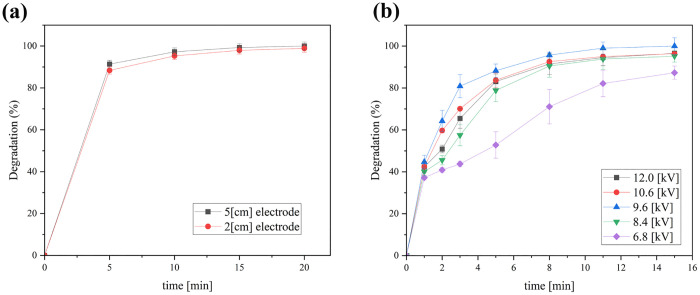
(a) Degradation efficiency dependence on Electrode size at 9.6 [kV] applied voltage for 20 mg/L MB solution, (b) Degradation efficiency for variation of applied voltages in HDC-DBD system for 20 mg/L MB solution.

Following preliminary degradation tests with distilled and tap water, the influence of applied voltage was systematically studied at 6.8, 8.4, 9.6, 10.6, and 12 kV (see Fig 9). Results showed that MB degradation rates at 9.6–12 kV were comparable, whereas at 6.8 and 8.4 kV, significantly lower removal efficiencies were observed during the initial 5 minutes (Fig 10(b)).

At voltages above 9.6 kV, excessive energy input caused localized heating, reducing the lifetime and density of reactive species. Conversely, voltages below 9.6 kV failed to generate sufficient plasma density for effective oxidation. Based on both OES data and concentration decay profiles, the 9.6 kV condition exhibited the most intense OH and O emission peaks and achieved the highest degradation efficiency. Therefore, 9.6 kV was selected as the optimal operating voltage for subsequent tests.

#### 3.1.7. Distilled water.

**Fig 11:**
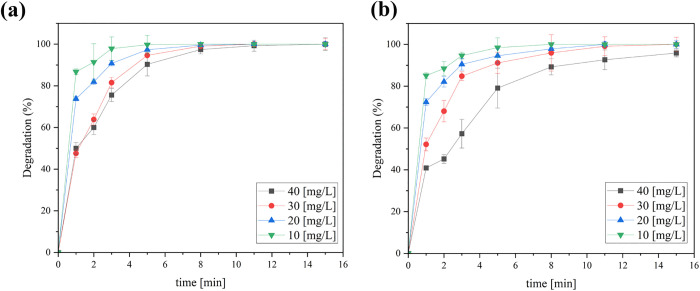
Concentration reduction profile of the MB solution prepared with (a) distilled, (b) tap water using HDC-DBD direct reactor over varying treatment durations.

For MB solutions prepared with distilled water, rapid and nearly complete degradation was observed (Fig 11(a)). The 10 mg/L solution reached 100% decolorization within 3 minutes. At 20 mg/L, the concentration decreased to 2.2% after 5 minutes and reached complete removal within 8 minutes. The 30 and 40 mg/L solutions exhibited similar behavior, achieving 100% degradation after approximately 8 and 14 minutes, respectively. These results confirm that plasma–cavitation synergy ensures efficient decomposition across various initial concentrations.

#### 3.1.8. Tap water.

**Fig 12:**
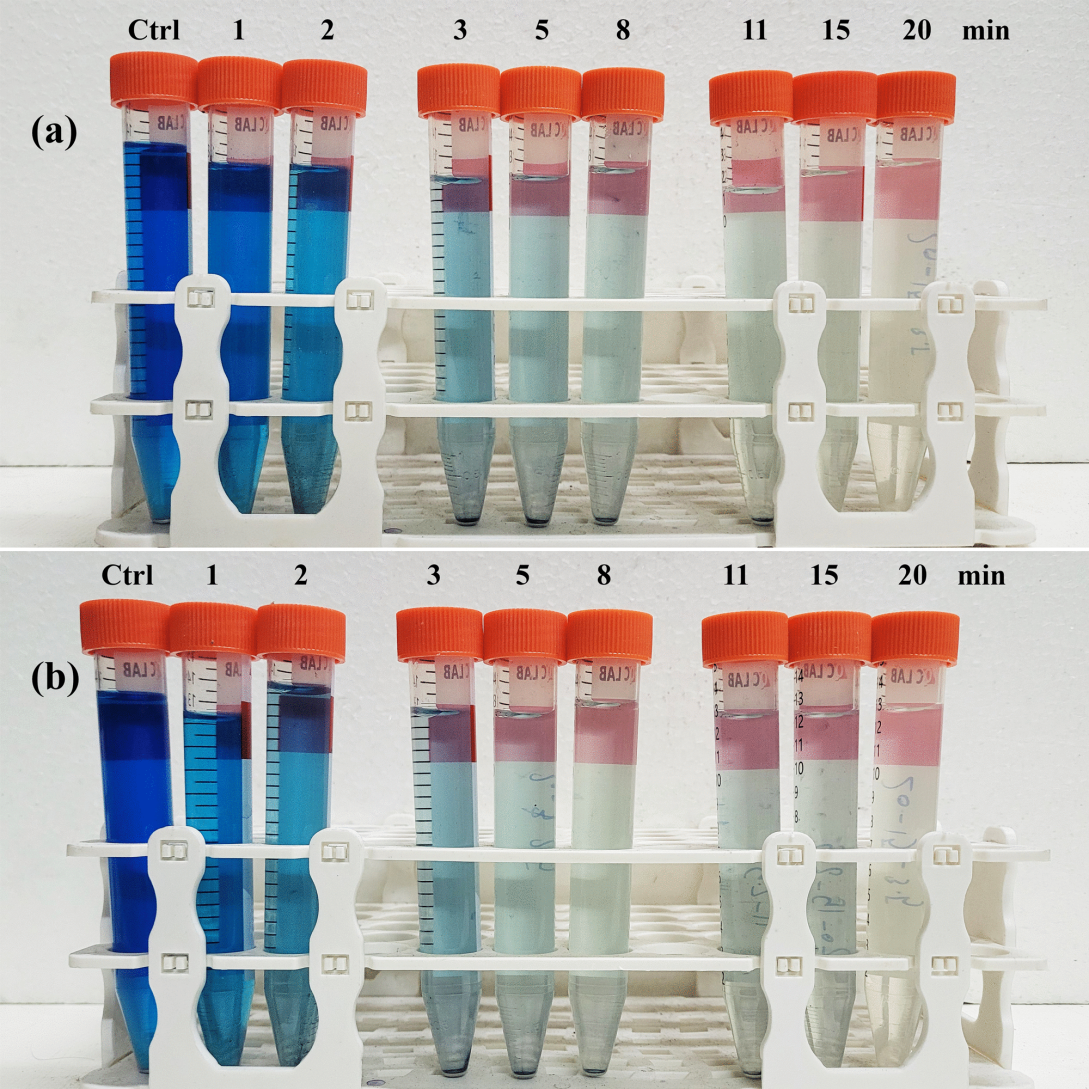
Comparative degradation of 40 mg/L MB solution prepared with (a) distilled water, (b) tap water at 1, 2, 3, 5, 8, 11, 15 and 20 minutes using direct DBD-HDC method (5 cm electrode).

Experiments conducted with tap water (Fig 11(b)) demonstrated slightly slower degradation due to the presence of background ions and buffering species. At 10 mg/L, MB concentration dropped by 99% within 3 minutes and reached complete removal after 4 minutes. For 20, 30, and 40 mg/L samples, degradation levels exceeded 97–100% within 15 minutes, confirming that DBD-HDC treatment remains highly effective even in mineralized water environments. Fig 12, demonstrates the degradation of MB in both distilled and tap water by direct HDC-DBD reactor (5 cm electrode).

#### 3.1.9. Comparative efficiency of direct and indirect DBD-HDC.

**Fig 13:**
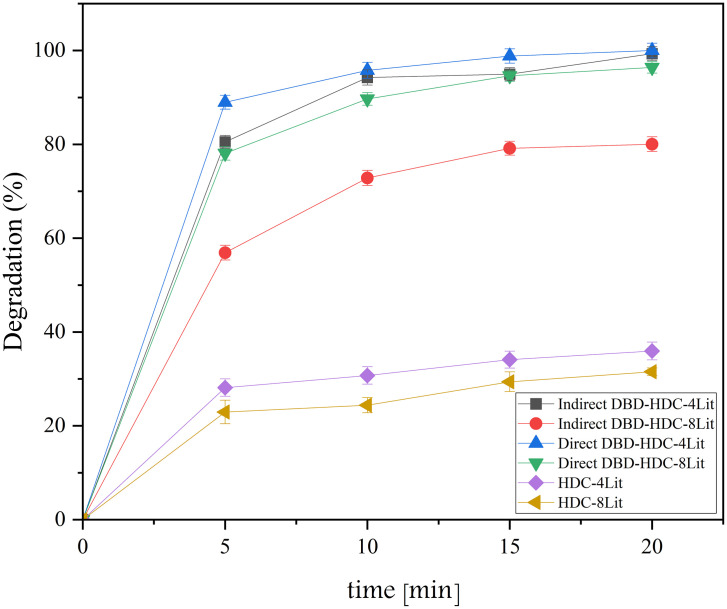
Comparative degradation analysis of 20 mg/L MB solution prepared with tap water in two volumes of 4 and 8 liters using direct and indirect method.

Comparative tests were performed using direct and indirect DBD-HDC reactor and also for HDC only at 4 L and 8 L volumes over 20 minutes (see Fig 13). In the indirect setup, degradation efficiencies reached approximately 95% and 78% for 4 L and 8 L systems, respectively. The direct reactor achieved ~99% and >95% degradation under the same conditions. The superior performance of the direct configuration is consistent with its higher OH radical intensity observed in OES spectra, confirming enhanced plasma–liquid interaction efficiency. These results demonstrate the scalability and enhanced reactivity of the integrated DBD-HDC system.

To substantiate the claimed synergistic interaction between DBD-HDC and HDC Only, control experiments were conducted under identical operational conditions. At an initial dye concentration of 20 mg/L and a reaction time of 20 min, HDC alone achieved a decolorization efficiency of 31.5% in the 8 L system and 36% in the 4 L system. These results indicate that although HDC contributes to pollutant degradation through cavitation-induced radical formation and micro-mixing effects, its standalone performance remains limited (< 40%).

In contrast, the combined DBD-HDC system exhibited significantly higher degradation efficiencies (as presented in Fig 13), clearly exceeding the removal achieved by HDC alone. This enhancement cannot be attributed merely to additive contributions of the two individual processes, suggesting the presence of a genuine synergistic interaction.

### 3.2. pH and EC analyses

#### 3.2.1. pH-distilled water solution.

**Fig 14:**
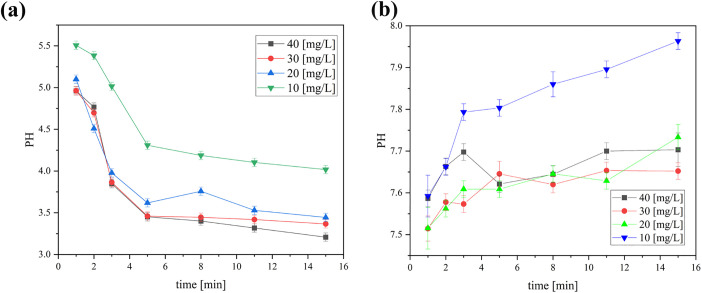
pH diagram of MB solution processed at concentrations of 10, 20, 30, and 40 mg/L, prepared with (a) distilled and (b) tap water.

In Fig 14(a), the pH exhibits a rapid decline during the initial minutes (0–4 minutes), indicating intense formation of NO and NO_2_ species at the early stages of plasma discharge, their prompt dissolution in water, and the rapid release of H⁺ ions from the resulting acids. As time progresses, the rate of pH decrease becomes more gradual. This can be attributed to the partial consumption of NO/ NO_2_, the approach of the system toward equilibrium, and, in some cases, the formation of basic species such as OH^-^ (derived from ∙OH radicals), which may contribute to stabilizing the pH [37].

#### 3.2.2. pH-tap water solution.

The initial pH of the tap water used for MB preparation was 7.63, and the addition of MB did not significantly alter the pH, indicating that MB does not introduce strong acidic or basic functionalities under the studied concentrations.

After plasma treatment, a slight but consistent increase in pH was observed, rising from approximately 7.55 to 7.65 after 15 minutes, with one sample reaching pH 8.

This behavior differs from that typically reported for distilled water systems, where plasma exposure commonly results in acidification due to the dissolution of NO_x_ species and subsequent formation of HNO_2_ and HNO_3_. The relatively stable or slightly increasing pH observed here can be attributed to the buffering capacity of tap water, primarily due to bicarbonate ions (HCO_3_^-^), which neutralize plasma-generated protons according to:


$$\begin{array}{c} H^{+}+HC\overset{-}{\underset{\text{3}}{O}}\to H_{\text{2}}CO_{\text{3}}\to CO_{\text{2}}+H_{\text{2}}O \end{array}$$ (9)

This buffering action mitigates significant pH reduction. Furthermore, under conditions where reactive oxygen species dominate over reactive nitrogen species, proton consumption and secondary reactions may lead to a slight net increase in pH [37].

The accompanying changes in EC further support the occurrence of plasma-induced aqueous chemistry, indicating the formation and redistribution of ionic species during treatment.

#### 3.2.3. EC-distilled water.

**Fig 15:**
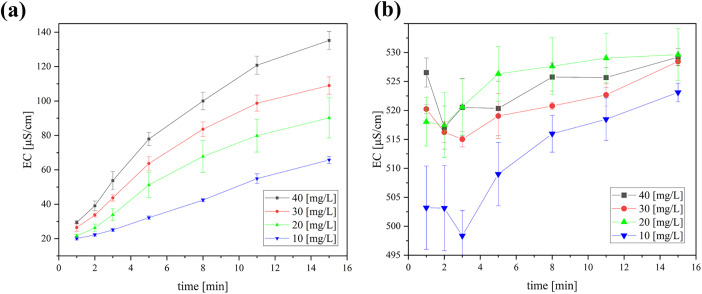
EC chart of MB solution processed at concentrations of 10, 20, 30, and 40 mg/L, prepared with (a) distilled and (b) tap water.

Fig 15(a) illustrates the evolution of EC as a function of plasma treatment time for MB solutions prepared with distilled water at varying concentrations (10, 20, 30, and 40 mg/L). The samples were subjected to DBD-HDC system for durations ranging from 0 to 15 minutes. A consistent and concentration-dependent increase in EC was observed over time across all tested MB concentrations.

Initially, all samples exhibit low EC values, consistent with the limited ionic content of the solutions prepared in distilled water. However, upon exposure to plasma, the EC begins to rise steadily, with the rate and magnitude of increase being more pronounced at higher dye concentrations. For instance, the 40 mg/L solution exhibits a sharp and continuous rise in conductivity, reaching approximately 130 μS/cm after 15 minutes, compared to the 10 mg/L solution, which plateaus near 70 μS/cm under the same conditions.

This increase in conductivity can be attributed to several plasma-induced physicochemical processes. Primarily, the DBD plasma generates a variety of RONS, such as H_2_O_2_, NO_3_^-^, NO_2_^-^ and H^+^, which are introduced into the solution and contribute significantly to its ionic strength. Additionally, plasma exposure induces degradation or oxidation of methylene blue molecules, leading to the formation of smaller ionic or polar fragments, further enhancing the solution’s EC.

Furthermore, at higher concentrations of MB, more dye molecules are available to undergo plasma-induced reactions, resulting in a greater generation of charged species. This explains the steeper conductivity increase observed for more concentrated solutions. It is also noteworthy that, unlike the case of tap water, the absence of background ionic species in distilled water allows the plasma effects to manifest more prominently in terms of conductivity enhancement. It should also be noted that the observed increase in EC in distilled water may partially originate from ion release from the electrodes during plasma treatment.

These findings confirm the capability of plasma treatment to significantly modify the ionic composition of MB solutions, especially when prepared in low-conductivity matrices like distilled water, and highlight the synergistic effect of plasma duration and dye concentration on EC [37].

#### 3.2.4. EC-tap water.

The presented Fig 15(b) illustrates the variation of EC of MB solutions over time for four different initial concentrations: 10, 20, 30, and 40 mg/L. At the beginning of the process (t = 0), the EC values increase with concentration, starting from approximately 506 μS/cm for 10 mg/L and reaching around 528 μS/cm for 40 mg/L. During the initial minutes (especially around 2 minutes), all concentrations experience a slight decrease or plateau in EC, which may be attributed to transient phenomena or the initial stabilization of the plasma-liquid interaction.

As time progresses, a gradual increase in EC is observed across all concentrations. For instance, the EC for the 40 mg/L sample rises from about 519 μS/cm at 2 minutes to approximately 530 μS/cm at 14 minutes. Similarly, the 20 and 30 mg/L samples reach around 528 and 526 μS/cm, respectively, by minute 15. The 10 mg/L sample, despite starting at the lowest value, also shows a steady increase, reaching approximately 520 μS/cm at the end of the treatment.

This upward trend in EC with time suggests the generation of conductive ionic species in the solution, likely due to the degradation of dye molecules into smaller, ionized fragments as a result of plasma treatment. The higher the initial concentration of the dye, the higher the final EC, indicating a concentration-dependent ion release. Overall, the increase in EC serves as an indirect indicator of the degradation efficiency and the formation of ionic byproducts during the plasma-assisted decolorization process [37].

#### 3.2.5. COD and TOC.

The mineralization performance of the cavitation system was evaluated through COD and TOC measurements. The initial methylene blue solution (40 mg/L) exhibited a COD of 64.6 mg O_2_/L and a TOC value of 23.7 mg/L. After 20 min of treatment, COD and TOC decreased to 15.1 mg O_2_/L and 7.3 mg/L, respectively.

These results correspond to a COD removal efficiency of 76.6% and a TOC reduction of 69.2%. The greater reduction observed in COD compared to TOC suggests rapid oxidation of organic intermediates and partial transformation into lower molecular weight compounds prior to complete mineralization. The substantial decrease in TOC confirms that the treatment process did not merely induce chromophore destruction or decolorization, but resulted in significant mineralization of the organic structure.

The difference between COD and TOC removal efficiencies indicates the progressive oxidation pathway typical of advanced oxidation processes, in which complex aromatic structures are first fragmented into smaller oxygenated intermediates before being fully converted into CO_2_ and H_2_O. The simultaneous reduction of both parameters demonstrates that the cavitation-driven system is effective not only in discoloration but also in the oxidative degradation of dissolved organic carbon.

#### 3.2.6. Energy utilization efficiency.

Table 1 presents the energy yield of MB degradation across various reference studies, highlighting the efficacy of the current work in achieving rapid degradation and enhanced MB elimination.

**Table 1:** Illustration of energy yield of MB degradation in variety of references.

P[W]	V [Lit]	treatment time[min]	C_0_[mg/L]	C[mg/L] eliminated	γ[g/kWh]	ref
8.6	0.1	30	100	10	2.32	[38]
5	–	40	50	–	–	[39]
18	0.05	20	100	5	0.83	[40]
206	0.05	25	50	2.5	0.03	[41]
–	0.1	40	100	7.7	–	[42]
6.3	0.03	40	35	1.05	0.25	[43]
10	0.1	20	150	13.5	4.05	[44]
8.6	0.1	30	100	9	2.09	[45]
150	0.3	20	12.5	3.75	0.07	[46]
139.3	4	15	40	160	4.6	This work

Most previous studies have focused on treating small volumes of contaminated water. In contrast, our research was conducted on substantially larger volumes, which inherently results in different quantities of pollutants at the same concentration across varying volumes. Consequently, this approach involves decomposing a greater total amount of dissolved contaminants compared to earlier works. This study introduces a new perspective: at an initial concentration of 40 mg/L, achieving complete (100%) destruction of the pollutant requires an energy consumption of 4.6 g/kWh. This represents a significant advancement in large-scale wastewater treatment, demonstrating lower energy consumption and enhanced efficiency.

**Fig 16:**
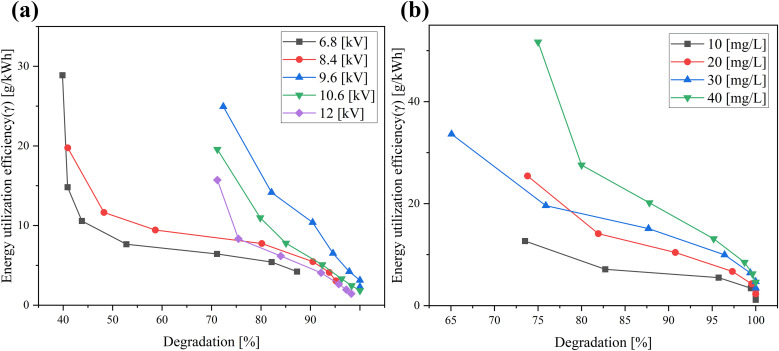
Energy utilization efficiency per degradation efficiency for (a) various applied voltages, (b) various initial concentrations.

Fig 16 illustrates the variation of the Energy utilization efficiency parameter (γ) with respect to degradation for different initial concentrations under a constant electrical discharge power of 139.3 W (9.6 kV). At approximately 80% degradation for 20 mg/L tap water solution, γ values ranges from 5 to 15 g/kWh across the tested initial concentration levels, demonstrating the system’s efficiency in achieving significant degradation within this range.

Fig 16(b) shows the dependence of γ on degradation for various input voltages, while maintaining a fixed initial concentration of 20 mg/L. At around 80% degradation, γ values lie between 10 and 30 g/kWh, indicating that voltage influences the process efficiency within this operational context.

### 3.3. Mechanistic interpretation of MB degradation under DBD-HDC treatment

To elucidate the degradation mechanism of MB in the hybrid DBD-HDC system, the generation and interaction of reactive species were analyzed. OES revealed prominent emission lines at 309 nm (•OH), 777 nm (O), and 337 nm (N_2_), indicating the simultaneous generation of RONS in the plasma zone. The observed decrease in pH and the concurrent increase in EC suggest the formation of oxidized ionic species such as HNO_2_, HNO_3_, and SO_4_^2-^, which enhance the ionic strength of the treated solution [47–49].

Exposure of MB solution to cold plasma in the presence of cavitation bubbles promotes decolorization through synergistic physical and chemical pathways. Reactive species generated by plasma interact with MB molecules, inducing oxidation-reduction reactions that disrupt the chromophoric structure and result in rapid decolorization. Both reactive oxygen species (ROS) and reactive nitrogen species (RNS) are produced in the discharge region, including primary radicals (H•, O•, OH•, N•, NO•) and secondary oxidants (H_2_O_2_, O_3_, NO_X_, HNO_2_, HNO_3_, ONOOH) [50].

Based on [51,52], major plasma-induced reactions include:


$$\begin{array}{c} O_{\text{2}}+e^{-}\to \text{2}O+e^{-} \end{array}$$ (10)

$$\begin{array}{c} N_{\text{2}}+e^{-}\to \text{2}N+e^{-} \end{array}$$ (11)

$$\begin{array}{c} N+O\to NO \end{array}$$ (12)

$$\begin{array}{c} O_{\text{2}}+O\to O_{\text{3}} \end{array}$$ (13)

$$\begin{array}{c} NO+O\to NO_{\text{2}} \end{array}$$ (14)

$$\begin{array}{c} O_{\text{3}}+NO\to NO_{\text{2}}+O_{\text{2}} \end{array}$$ (15)

$$\begin{array}{c} \cdot OH+\;\cdot OH\to H_{\text{2}}O_{\text{2}} \end{array}$$ (16)

$$\begin{array}{c} \cdot NO+\;\cdot OH\to HNO_{\text{2}} \end{array}$$ (17)

$$\begin{array}{c} N\overset{-}{\underset{\text{2}}{O}}+H^{+}\to HNO_{\text{2}}\; \end{array}$$ (18)

Hydroxyl radicals (•OH) generated in the plasma region can attack C = N and C = S bonds in MB, leading to chromophore disruption and color loss. Moreover, •OH can oxidize amino substituents on the aromatic ring to nitro or hydroxyl derivatives, further destabilizing the conjugated structure. O_3_ also acts as a strong electrophile, attacking electron-rich double bonds and enhancing the oxidative cleavage of MB [51,53].

The blue color of MB arises from its extended π-conjugated system centered on the phenothiazine ring, with an absorption maximum at 664 nm. Oxidative disruption of this conjugation eliminates its light-absorbing capability. MB also functions as a reversible redox indicator, interconverting between its oxidized (blue) and reduced (colorless) leucomethylene blue (LMB) forms. Under plasma exposure, free electrons and superoxide radicals reduce MB according to:


$$\begin{array}{c} C_{\text{1}\text{6}}H_{\text{2}\text{0}}N_{\text{3}}ClS\to \text{2}H^{+}+\text{2}e^{-}+C_{\text{1}\text{6}}H_{\text{1}\text{8}}N_{\text{3}}ClS \end{array}$$ (15)

Although this redox transition is theoretically reversible, continuous oxidative degradation by RONS prevents reoxidation to the blue form [54].

Prolonged plasma exposure further enhances oxidation, leading to thiazine ring cleavage, decomposition of aromatic intermediates (phenol, hydroquinone), and eventual mineralization into CO_2_, NH_3_, and SO_4_^2-^. Sequential oxidation of phenolic intermediates to benzoquinone, benzaldehyde, and ultimately CO_2_ and H_2_O has been reported under similar conditions [55]. The simultaneous decrease in pH and increase in EC observed experimentally thus correspond to the progressive formation of ionic and acidic end-products, confirming the synergy between plasma oxidation and hydrodynamic cavitation.

## 4. Conclusion

This study presents an innovative and effective hybrid approach integrating DBD plasma with hydrodynamic cavitation for the degradation of Methylene Blue in water. The results demonstrate that simultaneous operation of these processes yields superior performance and higher energy efficiency compared to their non-simultaneous counterparts. Complete decolorization (100%) for 40 mg/L solution was achieved under 15 minutes, with approximately 80% removal in less than 5 minutes.

Beyond simple decolorization, measurements of COD and TOC confirm significant and genuine mineralization of the organic pollutant, rather than merely destruction of the chromophore group. After 20 minutes of treatment 76.6% COD removal and 69.2% TOC reduction was obtained. The observed difference between COD and TOC removal efficiencies is consistent with the progressive oxidation pathway characteristic of advanced oxidation processes: complex aromatic structures are first rapidly fragmented into smaller oxygenated intermediates, which are then subsequently oxidized further and eventually fully mineralized to CO_2_ and water. This is a particularly important finding, as almost all other reported AOP technologies demonstrate very high decolorization rates, but very rarely achieve comparable levels of bulk organic carbon removal.

Notably, the energy utilization efficiency reached 4.6 g/kWh for the highest dye concentration (40 mg/L), with values ranging between 20–40 g/kWh across all tested concentrations. Comparative analysis indicates that this method surpasses existing technologies in all three core performance metrics: decolorization rate, extent of mineralization, and energy consumption. The experiments conducted on both distilled and tap water sources validate the robustness and applicability of this hybrid system under realistic operational conditions.

Overall, the proposed approach offers a promising, cost-effective, and environmentally friendly solution for wastewater treatment, particularly for the removal of dye pollutants. These findings address one of the most important and widely criticised limitations of competing advanced oxidation technologies, and underscore the significant potential for further development and industrial implementation of this system to enhance water purification processes and environmental sustainability.

## Supporting information

S1 File Raw experimental data for methylene blue concentration analysis. This file includes concentration calculation datasets for voltage-based experiments, direct DBD–HDC, tap water and distilled water systems, comparison between direct and indirect modes, electrode size effects, and raw pH and electrical conductivity (EC) measurements.
(XLSX)

S2 File Optical emission spectroscopy (OES) raw data. This file contains wavelength–intensity data for all applied voltages in both direct and indirect DBD–HDC configurations.
(XLSX)

S3 File Electrical power calculation data. Raw datasets used for determining discharge power at different applied voltages.
(XLSX)

S4 File Energy utilization efficiency and calibration data. Includes raw data used for calculating energy yield (g/kWh) across different voltages and initial concentrations, along with calibration curves for concentration determination.
(XLSX)

S5 File CFD simulation results. This file contains raw extracted data from CFD simulations, including velocity magnitude, absolute pressure distribution within the HDC unit, and axial pressure profiles along the flow path.
(XLSX)

S6 File Calculation of Table 1 contents. (XLSX)

S1 Video Plasma discharge operation video. Video recordings showing plasma formation and operation using 2 cm electrode under experimental conditions.
(MP4)

S2 Video Plasma discharge operation video. Video recordings showing plasma formation and operation using 5 cm electrode under experimental conditions.
(MP4)

S1 Text Complete list of captions and legends for all Supporting Information files. (TXT)

## References

[1] General Assembly Adopts Resolution Recognizing Access to Clean Water, Sanitation as Human Right, by Recorded Vote of 122 in Favour, None against, 41 Abstentions. 2010. https://press.un.org/en/2010/ga10967.doc.htm

[2] Cosgrove WJ, Loucks DP. Water management: Current and future challenges and research directions. Water Resources Research. 2015;51(6):4823–39. doi: 10.1002/2014wr016869

[3] Westall F, Brack A. The Importance of Water for Life. Space Sci Rev. 2018;214(2). doi: 10.1007/s11214-018-0476-7

[4] Al-Tohamy R, Ali SS, Li F, Okasha KM, Mahmoud YA-G, Elsamahy T, et al. A critical review on the treatment of dye-containing wastewater: Ecotoxicological and health concerns of textile dyes and possible remediation approaches for environmental safety. Ecotoxicol Environ Saf. 2022;231:113160. doi: 10.1016/j.ecoenv.2021.113160 35026583

[5] Baarimah AO, Bazel MA, Alaloul WS, Alazaiza MYD, Al-Zghoul TM, Almuhaya B, et al. Artificial intelligence in wastewater treatment: Research trends and future perspectives through bibliometric analysis. Case Studies in Chemical and Environmental Engineering. 2024;10:100926. doi: 10.1016/j.cscee.2024.100926

[6] Canter LW. Ground water pollution control. CRC Press. 2020.

[7] Sgroi M, Anumol T, Vagliasindi FGA, Snyder SA, Roccaro P. Comparison of the new Cl2/O3/UV process with different ozone- and UV-based AOPs for wastewater treatment at pilot scale: Removal of pharmaceuticals and changes in fluorescing organic matter. Sci Total Environ. 2021;765:142720. doi: 10.1016/j.scitotenv.2020.142720 33572038

[8] Liu Q, Zhu J, Ouyang W, Ding C, Wu Z, Ostrikov KK. Cold plasma turns mixed-dye-contaminated wastewater bio-safe. Environ Res. 2024;246:118125. doi: 10.1016/j.envres.2024.118125 38199474

[9] Angelina, Cullen PJ, Prescott SW, Leslie GL, Rao NRH, Henderson RK. A critical review on the application of non-thermal plasma bubbles for oxidation in water treatment. Chemical Engineering Journal. 2025;505:159667. doi: 10.1016/j.cej.2025.159667

[10] Mouele ESM, Tijani JO, Fatoba OO, Petrik LF. Degradation of organic pollutants and microorganisms from wastewater using different dielectric barrier discharge configurations--a critical review. Environ Sci Pollut Res Int. 2015;22(23):18345–62. doi: 10.1007/s11356-015-5386-6 26493299

[11] Yin Y, Xu H, Zhu Y, Zhuang J, Ma R, Cui D, et al. Recent Progress in Applications of Atmospheric Pressure Plasma for Water Organic Contaminants’ Degradation. Applied Sciences. 2023;13(23):12631. doi: 10.3390/app132312631

[12] Yawut N, Mekwilai T, Vichiansan N, Braspaiboon S, Leksakul K, Boonyawan D. Cold plasma technology: Transforming food processing for safety and sustainability. Journal of Agriculture and Food Research. 2024;18:101383. doi: 10.1016/j.jafr.2024.101383

[13] Tański M, Reza A, Przytuła D, Garasz K. Ozone Generation by Surface Dielectric Barrier Discharge. Applied Sciences. 2023;13(12):7001. doi: 10.3390/app13127001

[14] Seyfi P, Golghand MR, Ghasemi S, Ghomi H. The effect of mixed electric field on characteristic of ozone generation in a DBD plasma source. J Theor Appl Phys. 2020;14(3):195–202. doi: 10.1007/s40094-020-00385-2

[15] Wu M-C, Uehara S, Wu J-S, Xiao Y, Nakajima T, Sato T. Dissolution enhancement of reactive chemical species by plasma-activated microbubbles jet in water. J Phys D: Appl Phys. 2020;53(48):485201. doi: 10.1088/1361-6463/abae96

[16] Ning R, Yu S, Li L, Snyder SA, Li P, Liu Y, et al. Micro and nanobubbles-assisted advanced oxidation processes for water decontamination: The importance of interface reactions. Water Res. 2024;265:122295. doi: 10.1016/j.watres.2024.122295 39173359

[17] Ranade VV. Hydrodynamic Cavitation: Devices, Design and Applications. John Wiley & Sons. 2022.

[18] Sivakumar M, Pandit AB. Wastewater treatment: a novel energy efficient hydrodynamic cavitational technique. Ultrason Sonochem. 2002;9(3):123–31. doi: 10.1016/s1350-4177(01)00122-5 12154685

[19] Rajoriya S, Carpenter J, Saharan VK, Pandit AB. Hydrodynamic cavitation: an advanced oxidation process for the degradation of bio-refractory pollutants. Reviews in Chemical Engineering. 2016;32(4). doi: 10.1515/revce-2015-0075

[20] Kosel J, Gutiérrez-Aguirre I, Rački N, Dreo T, Ravnikar M, Dular M. Efficient inactivation of MS-2 virus in water by hydrodynamic cavitation. Water Res. 2017;124:465–71. doi: 10.1016/j.watres.2017.07.077 28800517

[21] Rajoriya S, Bargole S, Saharan VK. Degradation of reactive blue 13 using hydrodynamic cavitation: Effect of geometrical parameters and different oxidizing additives. Ultrason Sonochem. 2017;37:192–202. doi: 10.1016/j.ultsonch.2017.01.005 28427623

[22] Zezulka Š, Maršálková E, Pochylý F, Rudolf P, Hudec M, Maršálek B. High-pressure jet-induced hydrodynamic cavitation as a pre-treatment step for avoiding cyanobacterial contamination during water purification. J Environ Manage. 2020;255:109862. doi: 10.1016/j.jenvman.2019.109862 31778869

[23] Jyoti KK, Pandit AB. Water disinfection by acoustic and hydrodynamic cavitation. Biochemical Engineering Journal. 2001;7(3):201–12. doi: 10.1016/s1369-703x(00)00128-5

[24] Mancuso G, Langone M, Andreottola G. A critical review of the current technologies in wastewater treatment plants by using hydrodynamic cavitation process: principles and applications. J Environ Health Sci Eng. 2020;18(1):311–33. doi: 10.1007/s40201-020-00444-5 32399243 PMC7203374

[25] Gągol M, Przyjazny A, Boczkaj G. Wastewater treatment by means of advanced oxidation processes based on cavitation – A review. Chemical Engineering Journal. 2018;338:599–627. doi: 10.1016/j.cej.2018.01.049

[26] Badve MP, Bhagat MN, Pandit AB. Microbial disinfection of seawater using hydrodynamic cavitation. Separation and Purification Technology. 2015;151:31–8.

[27] Maršálek B, Zezulka Š, Maršálková E, Pochylý F, Rudolf P. Synergistic effects of trace concentrations of hydrogen peroxide used in a novel hydrodynamic cavitation device allows for selective removal of cyanobacteria. Chemical Engineering Journal. 2020;382:122383. doi: 10.1016/j.cej.2019.122383

[28] Abramov VO, Abramova AV, Cravotto G, Nikonov RV, Fedulov IS, Ivanov VK. Flow-mode water treatment under simultaneous hydrodynamic cavitation and plasma. Ultrason Sonochem. 2021;70:105323. doi: 10.1016/j.ultsonch.2020.105323 32911356 PMC7786523

[29] Pereira TC, Flores EMM, Abramova AV, Verdini F, Calcio Gaudino E, Bucciol F, et al. Simultaneous hydrodynamic cavitation and glow plasma discharge for the degradation of metronidazole in drinking water. Ultrason Sonochem. 2023;95:106388. doi: 10.1016/j.ultsonch.2023.106388 37011519 PMC10457580

[30] Foster J, Sommers BS, Gucker SN, Blankson IM, Adamovsky G. Perspectives on the Interaction of Plasmas With Liquid Water for Water Purification. IEEE Trans Plasma Sci. 2012;40(5):1311–23. doi: 10.1109/tps.2011.2180028

[31] Zaplotnik R, Primc G, Vesel A. Optical emission spectroscopy as a diagnostic tool for characterization of atmospheric plasma jets. Applied Sciences. 2021;11(5):2275.

[32] Kriegseis J, Möller B, Grundmann S, Tropea C. Capacitance and power consumption quantification of dielectric barrier discharge (DBD) plasma actuators. Journal of Electrostatics. 2011;69(4):302–12. doi: 10.1016/j.elstat.2011.04.007

[33] Abbas-Shiroodi Z, Sadeghi M-T, Baradaran S. Design and optimization of a cavitating device for Congo red decolorization: Experimental investigation and CFD simulation. Ultrason Sonochem. 2021;71:105386. doi: 10.1016/j.ultsonch.2020.105386 33232898 PMC7786587

[34] Raut-Jadhav S, Saharan VK, Pinjari D, Sonawane S, Saini D, Pandit A. Synergetic effect of combination of AOP’s (hydrodynamic cavitation and H_2_O_2_) on the degradation of neonicotinoid class of insecticide. J Hazard Mater. 2013;261:139–47. doi: 10.1016/j.jhazmat.2013.07.012 23912079

[35] Lamichhane P, Pourali N, Rebrov EV, Hessel V. Energy Intensified Nitrogen Fixation Through Fast Modulated Gas Discharge from Pyramid-shaped Micro-electrode. Plasma Chem Plasma Process. 2023;44(3):1369–92. doi: 10.1007/s11090-023-10376-1

[36] Maroofi A, Navab Safa N, Ghomi H. Atmospheric air plasma jet for improvement of paint adhesion to aluminium surface in industrial applications. International Journal of Adhesion and Adhesives. 2020;98:102554. doi: 10.1016/j.ijadhadh.2020.102554

[37] Bruggeman PJ. Plasma–liquid interactions: a review and roadmap. Plasma Sources Science and Technology. 2016;25(5):053002.

[38] Wu L, Xie Q, Lv Y, Wu Z, Liang X, Lu M, et al. Degradation of Methylene Blue via Dielectric Barrier Discharge Plasma Treatment. Water. 2019;11(9):1818. doi: 10.3390/w11091818 PMC9070396 35530987

[39] Chandana L, Manoj Kumar Reddy P, Subrahmanyam Ch. Atmospheric pressure non-thermal plasma jet for the degradation of methylene blue in aqueous medium. Chemical Engineering Journal. 2015;282:116–22. doi: 10.1016/j.cej.2015.02.027

[40] Wang B, Dong B, Xu M, Chi C, Wang C. Degradation of methylene blue using double-chamber dielectric barrier discharge reactor under different carrier gases. Chemical Engineering Science. 2017;168:90–100. doi: 10.1016/j.ces.2017.04.027

[41] Meiyazhagan S, Yugeswaran S, Ananthapadmanabhan PV, Suresh K. Process and kinetics of dye degradation using microplasma and its feasibility in textile effluent detoxification. Journal of Water Process Engineering. 2020;37:101519. doi: 10.1016/j.jwpe.2020.101519

[42] Marak FBFC, Joychandra Singh W, Mahanta D, Kapil N, Phanjom P, Loushambam HS, et al. Degradation of methylene blue through atmospheric pressure glow discharge plasma treatment. Phys Scr. 2023;99(1):015601. doi: 10.1088/1402-4896/ad14d2

[43] Abdel-Fattah E. Atmospheric pressure helium plasma jet and its applications to methylene blue degradation. Journal of Electrostatics. 2019;101:103360. doi: 10.1016/j.elstat.2019.103360

[44] Magureanu M, Piroi D, Gherendi F, Mandache NB, Parvulescu V. Decomposition of Methylene Blue in Water by Corona Discharges. Plasma Chem Plasma Process. 2008;28(6):677–88. doi: 10.1007/s11090-008-9155-x

[45] Wu L, Xie Q, Lv Y, Zhang Z, Wu Z, Liang X, et al. Degradation of methylene blue by dielectric barrier discharge plasma coupled with activated carbon supported on polyurethane foam. RSC Adv. 2019;9(45):25967–75. doi: 10.1039/c9ra05238k 35530987 PMC9070396

[46] Wang B, Sun B, Zhu X, Yan Z, Liu Y, Liu H. Degradation of Methylene Blue by Microwave Discharge Plasma in Liquid. Contrib Plasma Phys. 2013;53(9):697–702. doi: 10.1002/ctpp.201300007

[47] Shen J, Zhang H, Xu Z, Zhang Z, Cheng C, Ni G, et al. Preferential production of reactive species and bactericidal efficacy of gas-liquid plasma discharge. Chemical Engineering Journal. 2019;362:402–12. doi: 10.1016/j.cej.2019.01.018

[48] Bae JH, Huh S-C, Rahdar N, Park S. Real-Time Formation of Nitrate and Nitrite Species in Plasma-Activated Liquids: From Distilled Water to Cell Culture Solutions. Plasma Chem Plasma Process. 2026;46(2). doi: 10.1007/s11090-026-10638-8

[49] Zhao Z, Liu G, Li G, Ni W, Liu D. Reactive Oxygen and Nitrogen Species (RONS) Solubility Controlled Activation of Water by Atmospheric Pressure Air Spark Discharge. Plasma Chem Plasma Process. 2024;44(2):945–63. doi: 10.1007/s11090-024-10453-z

[50] He Y, Sang W, Lu W, Zhang W, Zhan C, Jia D. Recent Advances of Emerging Organic Pollutants Degradation in Environment by Non-Thermal Plasma Technology: A Review. Water. 2022;14(9):1351. doi: 10.3390/w14091351

[51] Du ChM, Sun YW, Zhuang XF. The Effects of Gas Composition on Active Species and Byproducts Formation in Gas–Water Gliding Arc Discharge. Plasma Chem Plasma Process. 2008;28(4):523–33. doi: 10.1007/s11090-008-9143-1

[52] Dorai R, Kushner MJ. A model for plasma modification of polypropylene using atmospheric pressure discharges. J Phys D: Appl Phys. 2003;36(6):666–85. doi: 10.1088/0022-3727/36/6/309

[53] Horňák R. Spatial mapping of OH radicals produced by electric discharge in hydrodynamic cavitation cloud. The Journal of Physical Chemistry Letters. 2025;16(25):6279–85. 40509855 10.1021/acs.jpclett.5c00979 PMC12207660

[54] Volkov AG, Hairston JS, Taengwa G, Roberts J, Liburd L, Patel D. Redox Reactions of Biologically Active Molecules upon Cold Atmospheric Pressure Plasma Treatment of Aqueous Solutions. Molecules. 2022;27(20):7051. doi: 10.3390/molecules27207051 36296644 PMC9608965

[55] Mvula E, von Sonntag C. Ozonolysis of phenols in aqueous solution. Org Biomol Chem. 2003;1(10):1749–56. doi: 10.1039/b301824p 12926365

